# A Pilot Study to Evaluate Pharmaceutical Pictograms in a Multispecialty Hospital at Dehradun

**DOI:** 10.4103/0975-1483.80306

**Published:** 2011

**Authors:** Y Joshi, P Kothiyal

**Affiliations:** *Himalayan Institute of Pharmacy & Research, Rajawala, India*; 1 *Faculty of Pharmacy, Dehradun Institute of Technology, Dehradun (Uttarakhand), India*

**Keywords:** Illiterate, interpretation, patients, pictograms

## Abstract

Pharmaceutical pictograms have the potential to play an important role in optimizing compliance in the illiterate patient population. Pictograms may improve warning comprehension for those with visual or literacy difficulties and can sometimes be recognized and recalled far better than words. The main purpose of this study is to determine whether these pictograms can be effectively understood by illiterate patients, who otherwise cannot read the instructions given on their prescription order. In this study, 10 pharmaceutical pictograms were evaluated in patients attending the outpatient department of the Shri Mahant Indresh Hospital, a multispecialty, referral hospital in Dehradun (Uttarakhand). Understanding of pictograms by patients before and after interpretation was noted and follow-up interpretation was also noted. Results of the study showed that prior to explanation, the majority of the patients were unable to interpret the pictograms correctly but after explanation of their meaning, interpretation by them showed a marked improvement, indicating the need of using pictograms along with verbal reinforcement. The study also highlighted poor patient follow-up, a major cause of patient non-compliance, often leading to a poor therapeutic outcome of the prescribed medication order. Such problems can be taken care of by an active participation by healthcare professionals.

## INTRODUCTION

The term pictogram is a collective term used to describe both ‘symbols’ and ‘pictorials’. Pictograms are standardized graphic images that help convey medication instructions, precautions, and/or warnings to patients and consumers. Pictograms are particularly helpful in passing on important information to patients with a lower level reading ability and for whom English is a second language.[1] Pharmaceutical pictograms have been designed to help people understand how to take their prescription medication. These are considered to be part of a universal language and can be easily recognized by all as they convey their meaning with little or no dependence on language or cultural background. Pictograms may improve warning comprehension for those with visual or literacy difficulties and can sometimes be recognized and recalled far better than words.[2] They have the potential to be interpreted more accurately and more quickly than words. It was shown that the presence of pictograms contributed positively to both understanding of instructions and adherence.[3] The success of using pictograms as a communication aid depends on a comprehensive design and testing process in order to produce clear, culturally acceptable pictograms, after which their value depends largely on their appropriate use by the healthcare professional who must provide verbal reinforcement in conjunction with the pictograms.[4,5]

## METHODOLOGY

**Figure 1:**
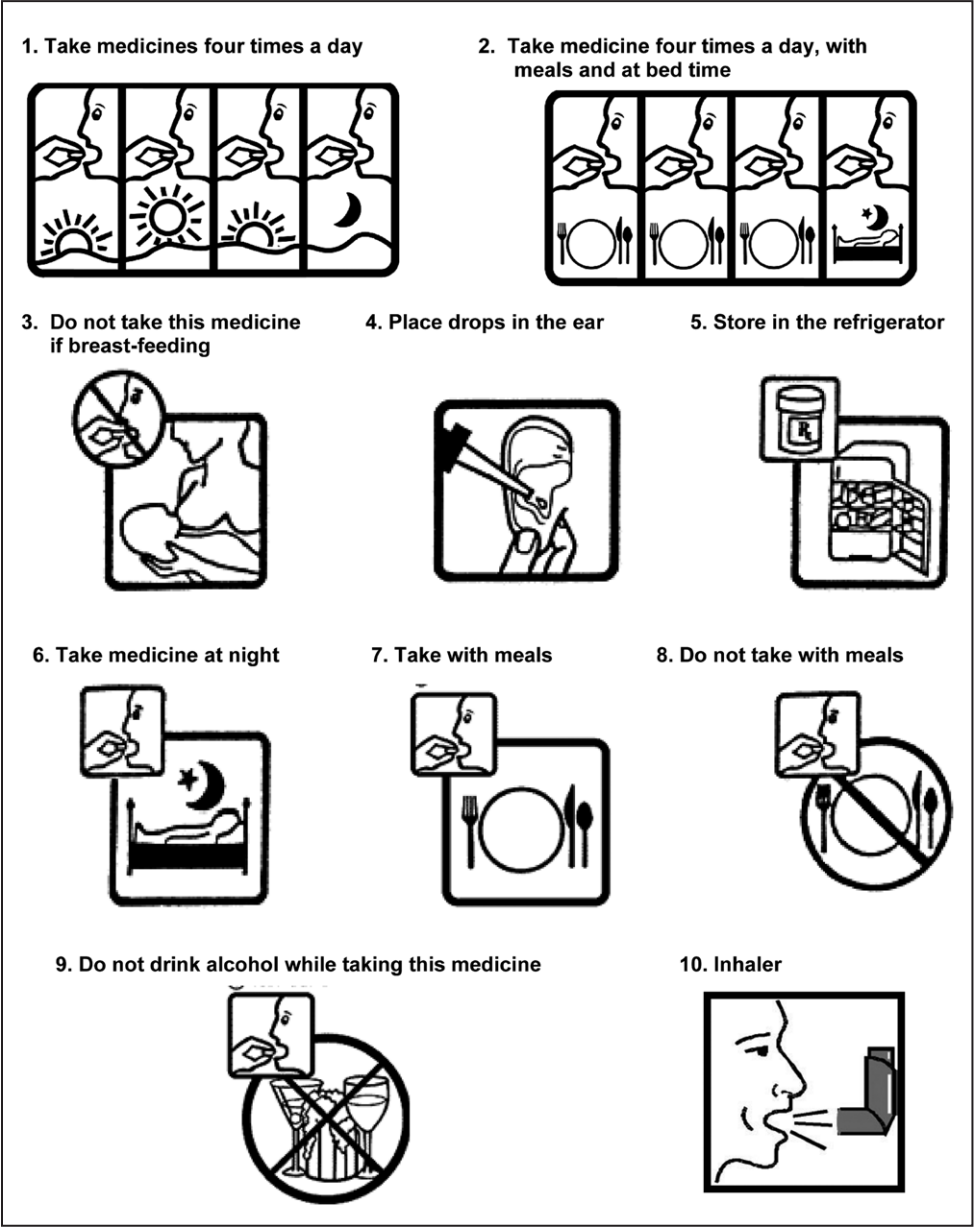
Pharmaceutical pictograms

The study was conducted at the outpatient department, Shri Mahant Indresh Hospital, a 300-bed multispecialty, referral hospital in Dehradun (Uttarakhand). A total of 200 patients agreed to participate in this study. All the patients chosen for the study were illiterate. Ten pharmaceutical pictograms were randomly selected for the study. Nine of these pictograms were sourced from the United States Pharmacopoeia Dispensing Information (USP-DI)[6] and the tenth pictogram was consciously selected keeping in mind the prevalence of asthma in Dehradun and poor usage of inhalers in such a population. Selected pharmaceutical pictograms were numbered from 1 to 10 and were given to the patients one by one to interpret their meaning. These 10 pictograms along with their meanings are shown in Figure 1. At the onset, patients were explained about the purpose of the study and invited to participate in it. The demographic details of the patients like name, gender, age, occupation and salary status were collected. Patients attending the outpatient department of the hospital were interviewed before and after explanation of the meaning of all 10 pictograms, and correct interpretation in percentage of pictograms was calculated accordingly. Follow-up interview of the patients was again planned according to their follow-up schedule in the prescription. Poor follow-up by the patients resulted in only 164 patients reporting back for an interview. Interpretation in percentage was calculated for these 164 patients.

## RESULTS

**Figure 2:**
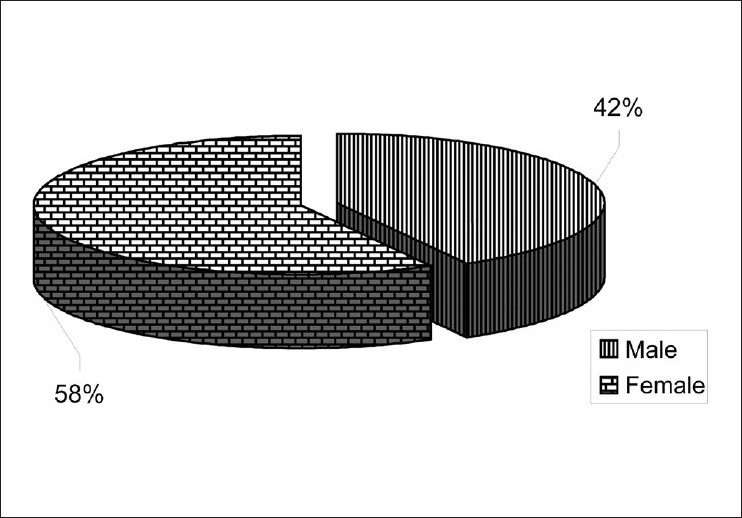
Gender-wise distribution of patients

**Figure 3:**
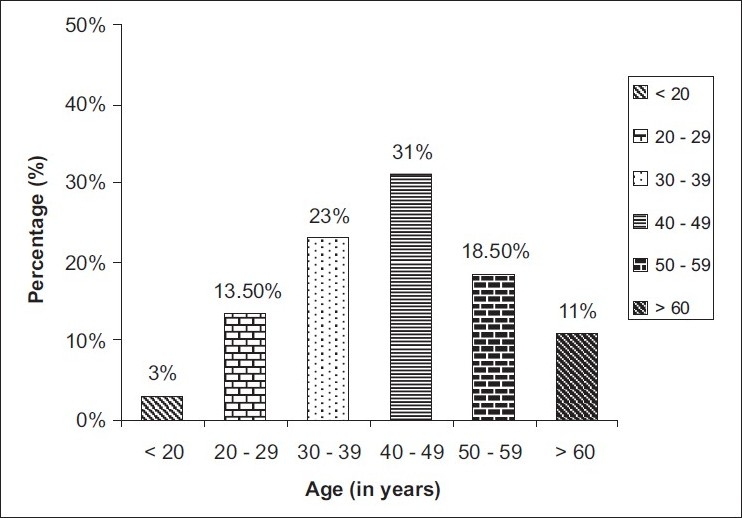
Age-wise distribution of patients

**Figure 4:**
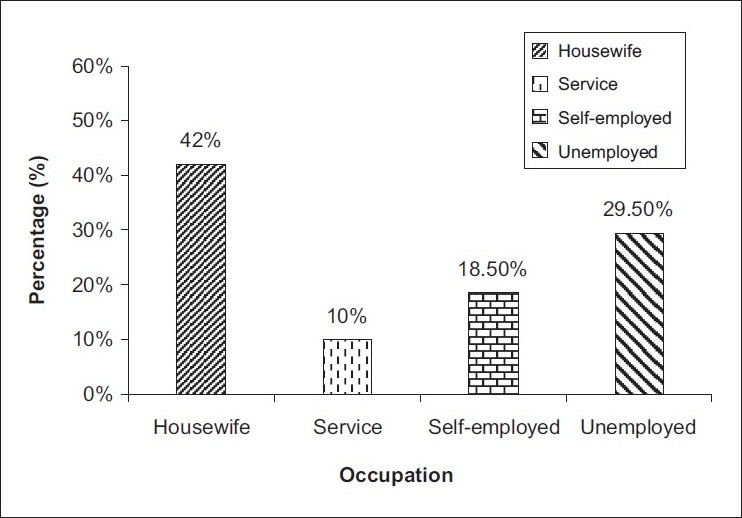
Occupation-wise distribution of patients

**Figure 5:**
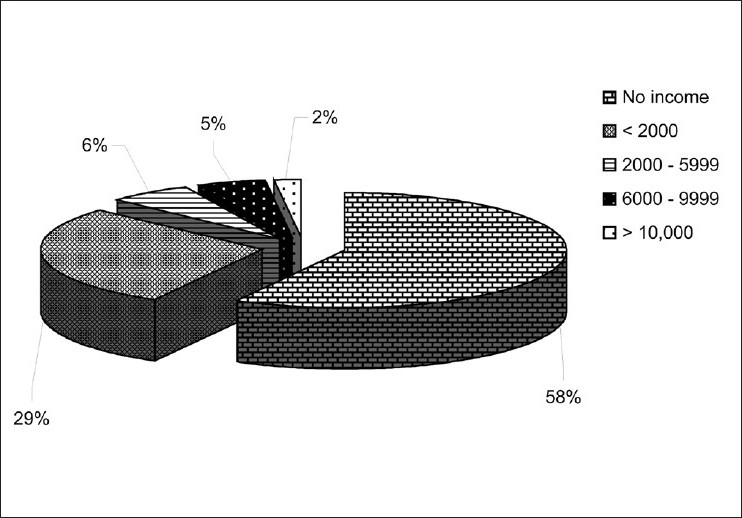
Salary-wise distribution of patients

**Figure 6:**
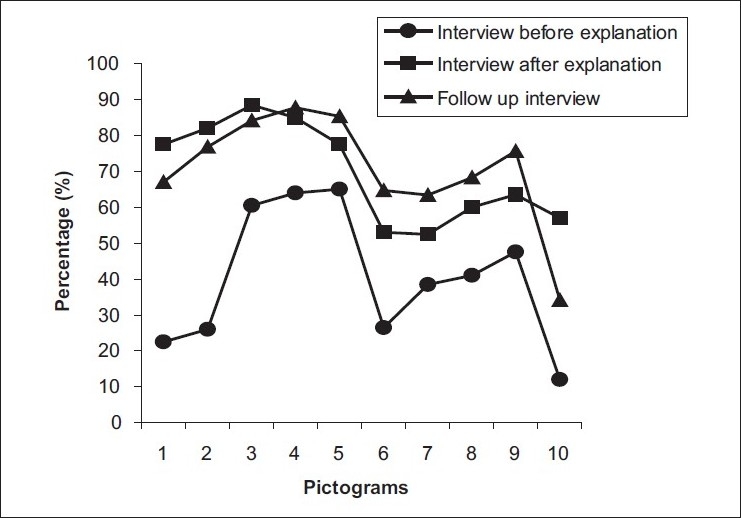
Comparison of interpretation of pictograms

The demographic details of the patients, viz. age, sex, occupation and salary are given in Table 1. There were 84 (42%) males and 116 (58%) females in our study [Figure 2]. The age-wise distribution of patients shows that the majority of the patients (31%) were between 40-49 years of age [Figure 3]. Occupation-wise distribution of patients showed that the majority of the patients (42%) were housewives [Figure 4]. Salary-wise distribution of patients showed that the majority of the patients (58%) were having no income [Figure 5]. Interpretation of pictograms before and after explanation as well as in the follow-up interview is depicted in Table 2 [Figure 6]. Before explaining the meaning of pictograms, the majority of the patients were unable to interpret them correctly but after explanation, interpretation shown by them comparatively improved. Prior to explanation, only 1% patients correctly interpreted the meaning of all 10 pictograms whereas after explanation 9.5% patients managed to interpret the meaning of all 10 pictograms. In the follow-up study, only 7.93% patients correctly interpreted the meaning of all 10 pictograms. Explanation comparatively improved the interpretation made by patients from 1% to 9.5% for all 10 pictograms. The most widely understood pictograms were – Take medicines four times a day, Place drops in the ear, Take medicine at night, Store in the refrigerator, Do not drink alcohol while taking this medicine and Do not take this medicine if breastfeeding.

**Table 1:** Demographic details of the patients

Distribution status	Number (%)
Gender	
Male	84 (42.00)
Female	116 (58.00)
Age (in years)	
< 20	06 (03.00)
20 - 29	27 (13.50)
30 - 39	46 (23.00)
40 - 49	62 (31.00)
50 - 59	37 (18.50)
> 60	22(11.00)
Occupation	
Housewife	84 (42.00)
Service	20 (10.00)
Self-employed	37 (18.50)
Unemployed	59 (29.50)
Salary (in Rs.)	
No income	116 (58.00)
< 2000	58 (29.00)
2000 - 5999	12 (06.00)
6000 – 9999	10 (05.00)
>10,000	04 (02.00)

**Table 2:** Correct interpretation of pictograms before and after explanation

Pictogram No.	First interview		Follow-up interview (out of 164 follow-ups)
	Before explanation	After explanation	
1	45 (22.50)	155 (77.50)	110 (67.07)
2	52 (26.00)	164 (82.00)	126 (76.83)
3	121 (60.50)	177 (88.50)	138 (84.15)
4	128 (64.00)	170 (85.00)	144 (87.81)
5	130 (65.00)	155 (77.50)	140 (85.37)
6	53 (26.50)	106 (53.00)	106 (64.64)
7	77 (38.50)	105 (52.50)	104 (63.42)
8	82 (41.00)	120 (60.00)	112 (68.29)
9	95 (47.50)	127 (63.50)	124 (75.61)
10	24 (12.00)	114 (57.00)	56 (34.15)

Value in parentheses indicates percentage (%)

## DISCUSSION

Illiterate patients cannot read the details in prescriptions, labels, leaflets and thus cannot remember instructions. Research has shown that the pharmaceutical pictograms were valuable if used in an appropriate manner, i.e. in combination with verbal and written reinforcement.[7,8] We, thus, evaluated selected pharmaceutical pictograms in the illiterate patient population of Dehradun. The majority of the patients were unable to interpret the meaning of pictograms correctly before explanation of their meaning but after explanation, interpretation of the meaning of pictograms comparatively improved. Only 1% patients correctly interpreted the meaning of all 10 pictograms prior to explanation whereas 9.5% patients managed to interpret the meaning of all 10 pictograms after the explanation of their meaning. Only 7.93% patients correctly interpreted the meaning of all 10 pictograms in the follow-up study and such poor patient follow-up during the study shows patient non-compliance, behavior which may ultimately affect the therapeutic outcome in them. Demographic information that showed the socioeconomic status of the study population included the key factors responsible for the outcomes of the study. The major emphasis to improve these limitations could be possible only by improving the literacy in such population.

## CONCLUSION

Prior explanation is required for proper recall of medication use instructions. Pictograms need to be developed for such illiterate patient population and the problem of patient non-compliance can be solved by the active participation by every healthcare professional with hand in hand cooperation by patients. However, it was felt that the pictograms need to be more culture-specific and can be reinforced with information, so as to cater to people of low socioeconomic strata. This would greatly enhance the patients’ understanding of the medication order and thus lead to improved compliance.
